# Intestinal short-chain fatty acid composition does not explain gut microbiota-mediated effects on malaria severity

**DOI:** 10.1371/journal.pone.0214449

**Published:** 2019-03-27

**Authors:** Shubham Chakravarty, Rabindra K. Mandal, Morgan L. Duff, Nathan W. Schmidt

**Affiliations:** Department of Microbiology and Immunology, University of Louisville, Louisville, Kentucky, United States of America; Food and Drug Administration, UNITED STATES

## Abstract

Malaria is a devastating disease resulting in significant morbidity and mortality, especially in the developing world. Previously, we showed that the gut microbiome modulates severity of malaria in mice, though the exact mechanism was unknown. One well-studied mechanism by which the intestinal microbiota exerts an effect on host health is by synthesis of short-chain fatty acids (SCFAs). SCFAs have pleiotropic effects on the host, including modulating the immune system and altering susceptibility to pathogens. The objective of the current work was to explore if gut microbiota-mediated resistance and susceptibility to malaria in mice is through differential production of SCFAs. Of the eight detected SCFAs, only propionic acid (C3) was different between two groups of resistant and two groups of susceptible mice, with higher levels in feces of susceptible mice compared to resistant mice. Nevertheless, subsequent analysis revealed no robust correlation between malaria severity and levels of fecal propionic acid. In spite of the broad effect of SCFAs on host physiology, including host immunity, this study shows that gut microbiota-mediated modulation of malaria severity in mice is independent of fecal SCFA levels. Additionally, our data indicates that intestinal SCFAs do not function as biomarkers for prediction of malaria disease severity.

## Introduction

*Plasmodium* infections remain a major health problem in tropical and sub-tropical countries, especially sub-Saharan Africa [1]. Therapeutic strategies to ameliorate the morbidity and mortality associated with malaria aim to reduce the severity of blood stage *Plasmodium* infections [2]. Previously we reported that the host gut microbiota can modulate the severity of *Plasmodium* yoelii 17XNL blood stage infections in C57BL/6 mice [3]. Genetically similar and age-matched mice from different commercial sources having different gut microbiota composition were shown to display differential susceptibility to blood stage parasite density and disease severity. Additionally, the resistant mice demonstrated a more robust humoral and cellular adaptive immune response in comparison to the susceptible mice. However, the mechanism determining resistance versus susceptibility to malaria severity has not been identified.

One well-studied mechanism by which intestinal microbiota alters host immunity and susceptibility to infections is by production of SCFAs [4–6]. Within the intestines, SCFAs regulate gut immune homeostasis by controlling pro- and anti-inflammatory response [7]. SCFAs foster an anti-inflammatory profile that results in a more tolerogenic immune profile by downregulating inflammatory mediators such as NF-KB, impeding pro-inflammatory cytokine production, and inhibition of T cell priming and activation by reducing expression of signaling molecules on dendritic cells (DCs) and other antigen presenting cells (APCs) [8]. SCFAs further support tolerance by increasing both the number and function of regulatory T cells. In this regard, SCFAs have been shown to increase expression of FOXP3 [9] as well as induce higher levels of the anti-inflammatory cytokine IL-10 [10]. Conversely, SCFAs promote protective pro-inflammatory immune responses in the gut. Mechanisms include promotion of increased intestinal epithelial barrier cell integrity [11] and inflammasome activation that increases production of pro-inflammatory cytokines such as IL-18 [12]. SCFAs elicit these pro- and anti-inflammatory effects by either binding to G protein-coupled receptors (GPCRs) and/or by acting as histone deacetylase (HDAC) inhibitors [7].

Production of SCFAs in the gut also impacts host immunity and host-pathogen interactions in extra-gastrointestinal tissues. SCFAs promote anti-inflammatory responses in the airways that limit immunopathology [13]. Recent reports also describe an important role for SCFAs in facilitating robust immune responses to clear viral and bacterial infections [4, 5]. Underlying mechanisms include modulating energy metabolism in immune cells by SCFAs, thereby promoting enhanced B and T cell responses.

Thus, given the well-established pleiotropic effects of SCFAs on host immunobiology, ultimately shaping the outcome of infectious diseases, the purpose of this present study was to investigate if there were differential abundances of SCFAs in the gut of mice resistant or susceptible to severe *P*. *yoelii* infections. Our results demonstrate that for most SCFAs, there is no differential fecal abundance between mice resistant or susceptible to severe *P*. *yoelii* infection. Even where there is a difference in abundance between vendors, such as propionic acid, there is no statistical correlation between fecal levels and parasite burden within vendor. Our data establishes that production of gut SCFAs is not a mechanism by which the gut microbiota modulates severity of *P*. *yoelii* infection in C57BL/6 mice and thus, SCFAs do not have the potential to serve as biomarkers to predict malaria disease severity.

## Materials and methods

### Mice

C57BL/6J female mice were purchased from The Jackson Laboratory (Jax) (Bar Harbor, Maine). C57BL6/N mice were purchased from Taconic Biosciences (Tac) (Hudson, NY), Charles River Laboratories (CR) (Wilmington, MD) and Envigo (Env) (Indianapolis, IN). Six-week-old mice from each vendor were delivered and allowed to acclimatize for one week before collecting fecal pellets and starting infections. Mice were kept on NIH-31 Modified Open Formula Mouse/Rat Irradiated Diet (Envigo) and autoclaved, non-acidified, reverse osmosis water. The mice were kept on a 12-hour light/dark cycle from 6 AM to 6PM and 6PM to 6AM, respectively. All animal handling and experimentation were reviewed and approved by the University of Louisville Institutional Animal Care and Use Committee based on the recommendations of the Guide for the Care and Use of Laboratory Animals of the National Institutes of Health. No analgesics were provided during the study. Mice were anesthetized with isoflurane prior to *P*. *yoelii* 17XNL infection for collection of blood via retro-orbital bleeding. One mouse within the Jackson Laboratory group did not recover from isoflurane treatment. There was no mortality following infection with *P*. *yoelii* 17XNL. Following infection mice were monitored by vivarium staff daily and laboratory personnel every other day during parasitemia analysis. Animal activity and body conditioning score were used to assess animal health and well-being. Mice were euthanized by carbon dioxide asphyxiation followed by cervical dislocation.

### Infections

Donor female Swiss Webster mice were infected with *Plasmodium yoelii* 17XNL intravenously. Fresh blood was collected from donor mice and experimental mice were subsequently infected with 10^5^ parasitized red blood cells (RBCs) via tail vein injection.

### Flow cytometry analysis of parasitemia

Blood was taken from tail snips on every alternate day from day 5 post infection (pi) and parasite burden was assessed by flow cytometry as described previously [3]. Briefly, 5 μl of blood was suspended in 1X PBS and fixed with 0.00625% glutaraldehyde in 1X PBS. This was followed by staining with the following: APC-CD45.2 (clone 104; Biolegend; San Diego, CA), APC/Cy7-Ter119 (clone TER-119; Biolegend; San Diego, CA), dihydroethidium (Sigma Aldrich; St. Louis, MO), and Hoechst 33342 (Sigma Aldrich; St. Louis, MO). APC/Cy7-Ter119^+^APC-CD45.2^-^ cell populations were identified as RBCs. Subsequently, RBCs were gated on Hoechst 33342 by dihydroethidium and Hoechst 33342^+^ plus Hoechst 33342^+^dihydroethidium^+^ RBCs were considered parasitized. Percentage parasitemia was calculated based on the percentage of RBCs that were found infected with *P*. *yoelii*.

### SCFA analysis from fecal pellets by gas chromatography (GC)-mass spectrometry (MS)

The fecal pellets from mice of all four groups were collected and immediately flash frozen in liquid nitrogen. SCFAs were quantified from fecal samples of mice as described previously [14] with a few modifications. The details of the experimental procedure are described below.

#### Chemicals and reagents

SCFA standards (sodium formate, sodium acetate, sodium propionate, sodium butyrate, isobutyric acid, sodium pentanoate (sodium valerate), 2-methylbutanoic acid, and isovaleric acid) and 2, 3, 4, 5, 6-pentafluorobenzyl bromide (PFBBr) were purchased from MilliporeSigma (St. Louis, MO, USA)

#### Preparation of standard solution for external calibration curve

Standard solutions that mix the same amount of each SCFA standard were prepared in 7 concentrations: 5 μM, 10 μM, 25 μM, 50 μM, 100 μM, 250 μM and 500 μM. 100 μL of each standard solution was collected for derivatization.

#### Preparation of fecal samples

All fecal samples were processed on ice to minimize volatile SCFAs loss, unless stated otherwise. Deionized water was added into fecal samples at a ratio of 20 μL water/1 mg feces. The mixture was sonicated for 20 min and then centrifuged at 4°C, 12,000 rpm for 20 min. 100 μL of supernatant was collected for derivatization.

#### Derivatization

100 mM PFBBr in acetone, 0.15 M phosphate buffer (PBS, pH 7.0) and standard solution/sample supernatant were mixed at a ratio of 14:2:5 (v:v:v) in 2 mL glass tubes. After 2 min of vigorous vortexing, the mixture was incubated in a water bath at 60°C for 1.5 h. Once the mixture cooled to room temperature, 100 μL hexane was added followed by 3 min vortex and 5 min centrifugation. 90 μL of the upper layer (hexane phase) was transferred into a 200 μL GC vial for GC–MS analysis.

#### GC–MS analysis

GC-MS analysis was carried out on Thermo Scientific ITQ 1100 GC-Ion Trap MS coupled with TRACE 1310 gas chromatography and 1310 autosampler (ThermoFisher Scientific, Waltham, MS, USA). Agilent J&W DB-225ms (30 m × 0.25 mm i.d., 0.25 μm film thickness, Santa Clara, CA, USA) and Agilent J&W DB-5ms (30 m × 0.25 mm i.d., 0.25 μm film thickness, Santa Clara, CA, USA) were hyphenated by a column connector for GC separation. Operating conditions were as follows: The injection volume was 1 μL in split mode at a split ration of 10; the carrier gas was helium at a constant flow of 1.0 mL/min; the temperatures of inlet, ion source and transfer line were all 220°C; the oven temperature programmed was 40°C for 0.5 min, 10°C/min to 158°C and hold for 0.5 min, 3°C/min to 160°C and hold for 0.5 min, 20°C/min to 220°C and hold for 5.0 min. Analysis was performed in the selected ion monitoring mode (Table 1):

**Table 1:** Ion monitoring mode for individual short-chain fatty acids.

Compound name	Monitored ion *m/z*	Monitor region (min)
Formic acid	181, 226	13.90–14.45
Acetic acid	181, 240	14.45–15.20
Propionic acid	181, 254	15.20–15.90
Isobutyric acid	181, 268	15.90–16.90
Butyric acid	181, 268	15.90–16.90
2-methylbutanoic acid	181, 282	16.90–22.00
Isovaleric acid	181, 282	16.90–22.00
Valeric acid	181, 282	16.90–22.00

#### Data processing

Thermo Xcalibur software (2.2 SP1.48) was used to process the GC–MS data. The external calibration curve was linear curve with 1/x weighting. The concentration of SCFA in a biological sample was calculated by fitting the external calibration curve.

### Graphical and statistical analysis

All graphical and statistical analyses of data were done using version 6 of GraphPad Prism. The area under the percent parasitemia curve (AUC) was calculated as previously reported [3, 15] using the following equation: AUC_t1-t-last_ = 0.5 Σ (Y_i_ + Y_i+1_) * (t_i+1_-t_i_); here “t” represents sampling time and “y” is the observed outcome (i.e., % parasitemia). For details of statistical analysis refer to figure legends.

## Results

### Minimal differences in fecal SCFA levels in mice differentially susceptible to *P*. *yoelii*

**Fig 1:**
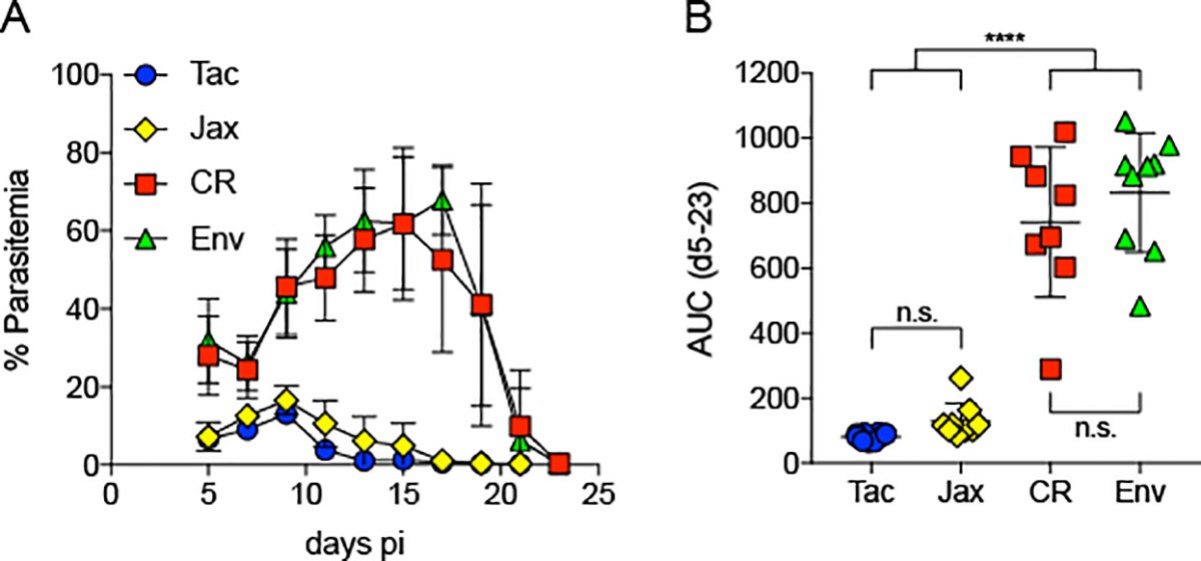
Analysis of *P*. *yoelii* parasitemia in mice from different vendors. A) Percentage (out of total RBCs) of infected RBCs. B) AUC analysis. Data were obtained from 10 mice from each vendor, except for Jax (n = 9). Data were analyzed by ordinary one-way ANOVA test followed by Tukey’s multiple comparison test. *p*<0.05 was considered statistically significant.

**Fig 2:**
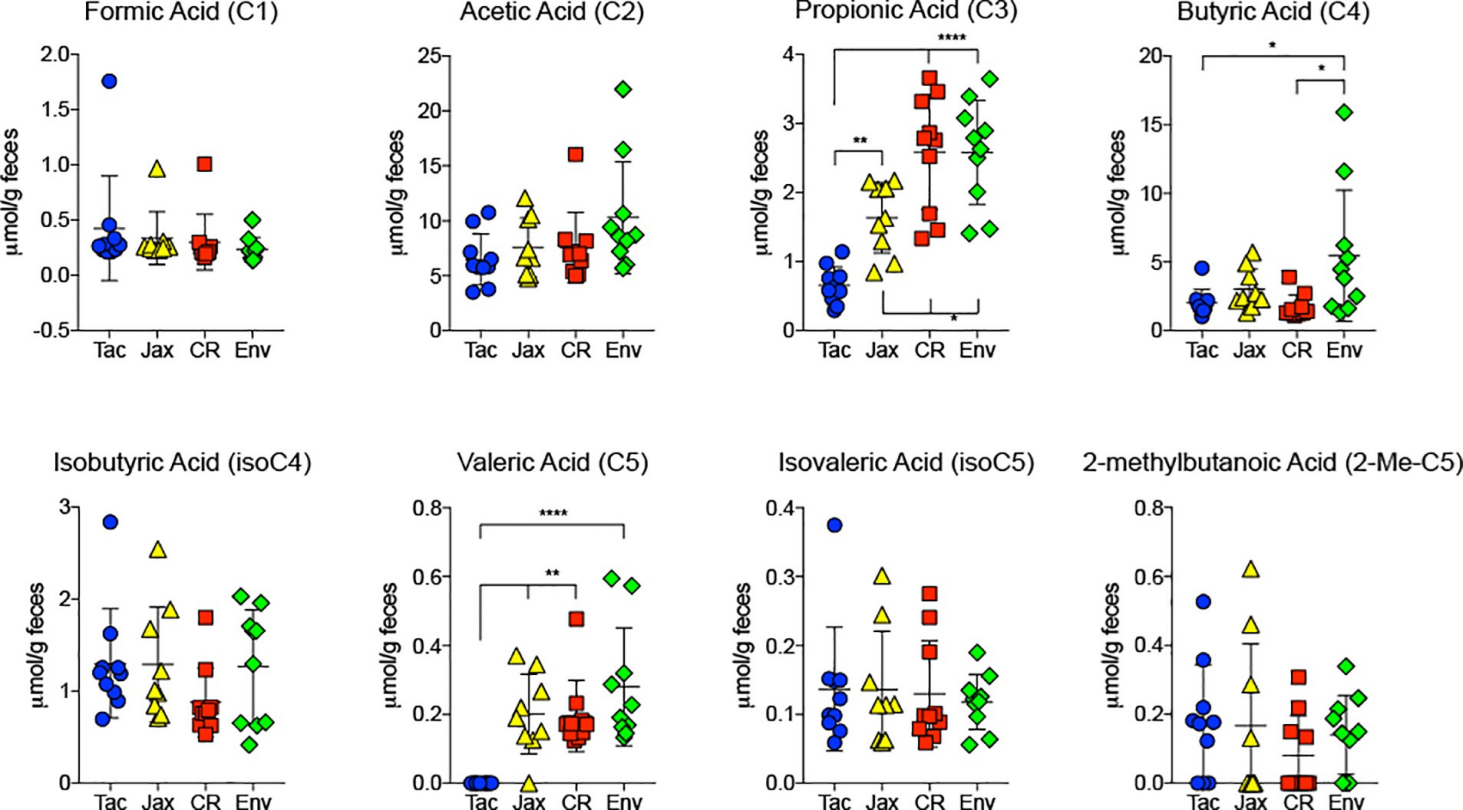
SCFA quantification in the feces of mice from different vendors. Fecal pellets were collected from 10 mice from each vendor, except for Jax (n = 9). Data were analyzed by ordinary one-way ANOVA test followed by Tukey’s multiple comparison test. *p*<0.05 was considered statistically significant.

We have previously shown that mice from Taconic Biosciences (Tac) and Jackson Laboratory (Jax) are resistant to severe *P*. *yoelii* infection, while mice from Envigo (Env) and Charles River Laboratories (CR) were susceptible [15]. These resistant and susceptible phenotypes were shown to be solely driven by the gut microbiota composition of the mice from the respective vendors. Given the beneficial effects of SCFAs on host immunity to infectious disease, we hypothesized that there would be an increase in SCFAs in the intestinal contents of mice resistant to *P*. *yoelii* as compared susceptible mice. To test this, we collected fecal pellets from resistant and susceptible mice and analyzed the contents for SCFAs by targeted GC-MS [14]. Subsequently, we infected the mice and monitored infection kinetics and parasite burden to confirm that the mice displayed the correct phenotypes. Consistent with our previous finding, mice from Tac and Jax vendors exhibited much lower parasite burden, compared to mice from CR and Env (Fig 1A and 1B). Subsequent analysis of SCFAs from fecal pellets collected from these mice prior to infection revealed limited differences in the two groups of resistant mice (Tac and Jax) when compared to susceptible mice (Env and CR) (Fig 2). Out of the eight SCFAs tested, formic acid, acetic acid, isobutyric acid, isovaleric acid and 2-methylbutanoic acid were found in similar levels in the feces of resistant and susceptible mice. In contrast, fecal levels of propionic acid were found to be significantly higher in susceptible mice (CR and Env) in comparison to resistant mice (Tac and Jax). Additionally, levels of propionic acid were significantly higher in feces of Jax mice compared to Tac mice. Valeric acid levels were also higher in both groups of susceptible mice (CR and Env) in comparison to Tac but not to Jax mice (Fig 2). Also, there was a significantly higher abundance of valeric acid in the feces of Jax mice compared to Tac mice. Taken together, there are minimal differences in SCFA levels between C57BL/6 mice resistant or susceptible to severe malaria.

### Intestinal propionic acid levels do not correlate with malaria severity

**Fig 3:**
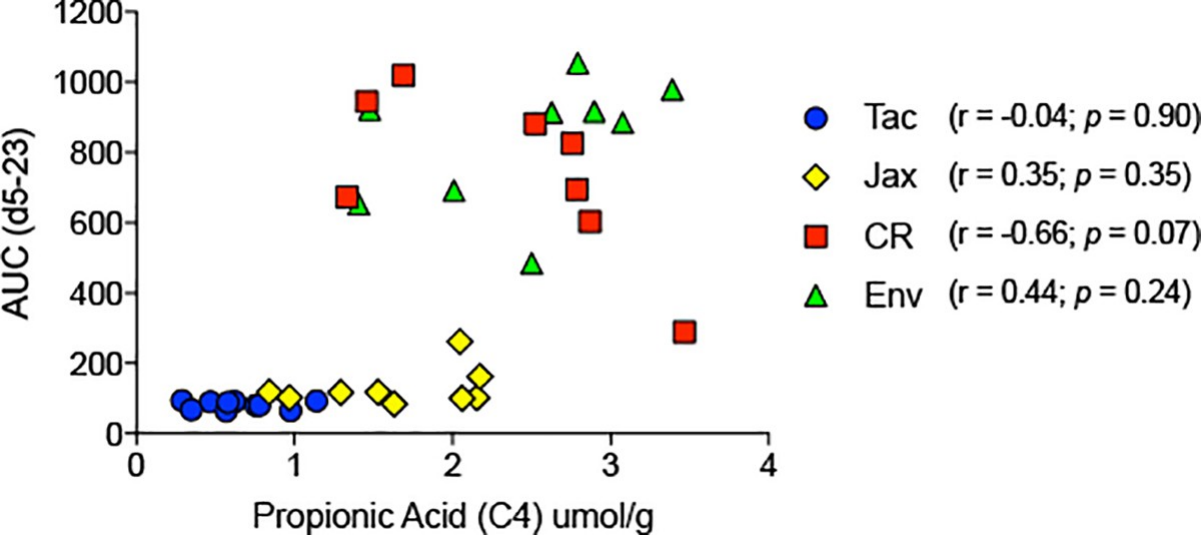
Correlation analysis between propionic acid levels in the feces of mice and corresponding parasite density. Pearson’s coefficient was calculated, followed by estimation of two-tailed *p* values.

As shown in Fig 2, propionic acid levels were higher in the feces of both groups of susceptible mice (CR and Env) in comparison to both groups of resistant mice (Tac and Jax). Additionally, SCFAs have been reported to mediate anti-inflammatory responses in the host [16–18]. Specifically, a recent report describes the anti-inflammatory properties of propionate [19]. We thus wanted to test the hypothesis that increased propionic acid levels in the gut conferred susceptibility of CR and Env mice to *P*. *yoelii* blood stage infection. To this end, we performed a correlation analysis on individual mice from all four groups to see if increased intestinal levels of propionic acid indeed correlated with greater parasite burden. As shown in Fig 3, the Pearson r values for Tac, Jax, and Env demonstrated no strong correlation between parasite burden (AUC) and propionic acid level. Of note, the r value with the highest magnitude, which approached statistical significance, was the CR group that actually had a negative correlation between parasite burden and level of propionic acid. Thus, no correlation could be drawn between intestinal propionic acid levels and severity of *P*. *yoelii* blood stage infection in C57BL/6 mice.

## Discussion

A previous study by our group identified that the gut microbiota in mice is a factor determining resistance or susceptibility to severe malaria in the host [3], but the mechanism for this observed effect is unknown. This current work establishes that differential production of intestinal SCFAs by the gut microbiota is not a mechanism behind this phenotype. Whereas these data demonstrate that the gut microbiota does not differentially modulate SCFA abundances as a mechanism to confer resistance to severe malaria, it is unknown what effect prophylactic treatment with SCFAs would have on the severity of malaria given the immunomodulatory roles of these molecules.

It is surprising that higher SCFA abundances, particularly of butyrate, were not seen in the fecal pellets of resistant mice. While SCFAs generally have been attributed with anti-inflammatory-mediating effects in the host [13, 20], a recent report described the beneficial role of butyrate in conferring immunity to influenza virus [4]. Moreover, butyrate has been implicated in facilitating a robust humoral immune response in the host, by altering catabolic processes in immune cells [5, 6] and modulating gene expression by inhibition of HDACs leading to differentiation of plasma B cells [21].

Although there was no correlation with parasite burden (Fig 3), significant differences were observed in SCFA levels between the resistant and susceptible mice groups. In fact, subtle SCFA composition differences were observed even between each group of resistant mice (seen in propionic and valeric acid). Given that mice from all four vendors were genetically similar, fed the same diet, and housed in identical conditions, this highlights the disparity in gut microbiota compositions between these groups, as we previously reported [3]. Although not affecting the severity of malaria, it would be intriguing to know what effect the differential abundance of propionic and valeric acid in these different groups of mice (Fig 2) have on other aspects of immunity and inflammation (e.g., autoimmunity or allergy). As an interesting example, a recent report [22] described how gut microbiota produced propionate confers resistance to *Salmonella* infections.

Taken together, our current study establishes that alteration of intestinal SCFA abundance is not a mechanism by which gut microbiota impacts blood stage malaria severity. As a corollary, since intestinal SCFA profiles were not found to correlate with malaria severity, fecal SCFA signatures are not candidates to serve as biomarkers for susceptibility to severe malaria. In future studies it is imperative to examine whether other metabolites or microbial components explain how the gut microbiota shapes severity of malaria in the host.
