# Correction: Intestinal short-chain fatty acid composition does not explain gut microbiota-mediated effects on malaria severity

**DOI:** 10.1371/journal.pone.0218250

**Published:** 2019-06-06

**Authors:** Shubham Chakravarty, Rabindra K. Mandal, Morgan L. Duff, Fang Yuan, Xiang Zhang, Nathan W. Schmidt

Fang Yuan and Dr. Xiang Zhang are not included in the author byline.

Fang Yuan should be listed as the fourth author, and her affiliation is: the Department of Chemistry, University of Louisville, Lousioville, Kentucky, Unived States of America. Her contribution is as follows: Investigation.

Xiang Zhang should be listed as the fifth author, and his affiliation is: the Department of Chemistry, University of Louisville, Lousioville, Kentucky, Unived States of America. His contributions is as follows: Resources.

The correct citation is: Chakravarty S, Mandal RK, Duff ML, Yuan F, Zhang X, Schmidt NW (2019) Intestinal short-chain fatty acid composition does not explain gut microbiota-mediated effects on malaria severity. PLoS ONE 14(3): e0214449. https://doi.org/10.1371/journal.pone.0214449
